# Listening to the experts: Parents' perspectives around infection risk and returning to education and social activities following their child's diagnosis of acute lymphoblastic leukemia

**DOI:** 10.1002/cnr2.1424

**Published:** 2021-05-14

**Authors:** Kirsten Ballantine, Claire Gooder, Elizabeth Ryan, Scott Macfarlane

**Affiliations:** ^1^ National Child Cancer Network Auckland New Zealand; ^2^ Children's Haematology Oncology Centre Christchurch Hospital Christchurch New Zealand; ^3^ Child Cancer Foundation Auckland New Zealand; ^4^ Starship Blood and Cancer Centre Starship Child Health Auckland New Zealand

**Keywords:** communication, decision‐making, infection‐risk, parental, school

## Abstract

**Background:**

During a child's prolonged treatment for acute lymphoblastic leukemia (ALL), there is a need to balance their increased risk of developing infection‐related complications with meeting their educational and social needs.

**Aims:**

To determine the safe timing of return to social activities for children undergoing treatment for ALL and to determine how parents perceive and act on advice related to infection risk while navigating their child's “return to normal.”

**Methods and results:**

Medical and educational attendance records were reviewed for 47 children who were diagnosed with ALL and 24 semi‐structured qualitative interviews were conducted with a representative sample of their parents. The majority of children (69%) did not return to education prior to the start of maintenance therapy regardless of the advice that the families received from their healthcare team. Those who returned earlier were at no greater risk of major infection complications (mean = 0.5) than those who did not return until after commencing maintenance (mean = 0.4, *P* = .74). Parents spoke of the difficulty in obtaining practical, consistent, and timely advice and of balancing infection risk with a desire to return to normalcy. Inconsistent advice and constant vigilance placed a burden on parents which often profoundly affected their mental wellbeing. Overall, parents wanted to make their own decisions about how and when their child returned to education and social activities. They made these decisions based on many factors, of which infection risk was just one.

**Conclusion:**

Following the study conclusion, a national working group was established—including parent representatives—to implement the study recommendations. This includes the development of a range of practical resources to better support families. Health professional guidelines provide quantitative data pertaining to infection risk, while emphasizing that the returning decisions ultimately rest with the families. This research demonstrates that listening to parents—who are the experts through their lived experiences—is a critical element in creating policies that are responsive, meaningful, and widely accepted.

## 1 INTRODUCTION

Acute lymphoblastic leukemia (ALL) is a disease of the blood‐forming tissues of the bone marrow, which can occur at all ages, though it is primarily a disease of childhood.^1^ ALL accounts for over one in four of all new cases of cancer in New Zealand children each year and currently has a 5‐year survival rate of over 90%.^2^ The long course of immunosuppressive chemotherapy treatment—typically 2–3 years—means that there is an extended period of risk of infection‐related complications, which can lead to changes in treatment, deferral or interruption of a treatment phase, and re‐hospitalisation.^3^, ^4^, ^5^ Infections occur across the whole treatment period, with some infections more common during the induction phase,^3^, ^6^ but higher numbers of infections documented during the longer maintenance phase.^5^ Infection‐related complications can lead to costly additional hospital admissions, resulting in further stress and upheaval for the child and their family.

For parents, caregivers and health professionals, there is a desire to find a balance between the risks of infection for children undergoing treatment for ALL and meeting the child's social and educational needs. The return to social activity parent‐directed resources and the academic literature have largely focused on *why* and *how*,^7^, ^8^, ^9^, ^10^ with the implication that returning to education and social activities should happen as soon as possible due to the psychosocial benefits for the child as well as the wider family considerations such as parents' ability to return to paid work.^11^, ^12^ Despite these recognized benefits, medical professionals—even within the same workplace—have been shown to hold differing opinions about infection risk and the timing of return to social activities.^13^, ^14^ There is currently no existing body of research that has determined an optimal approach with regard to *when* it is safe for children undergoing treatment for cancer to return to education and other social activities.^14^

All children diagnosed with cancer in New Zealand have their care coordinated by a specialist multidisciplinary child cancer team based at either the Children's Haematology Oncology Centre (CHOC) in Christchurch or Starship Blood and Cancer Centre (SBCC) in Auckland. These two specialist pediatric oncology treatment centers work closely with 14 shared‐care pediatric teams so that patients can receive as much of their care as close to home as is safely possible. The child's induction phase of treatment is undertaken at one of the two specialist centers, while the remainder of their treatment can predominantly be administered at the child's local shared care hospital, with additional scheduled appointments at their specialist center.

**FIGURE 1:**
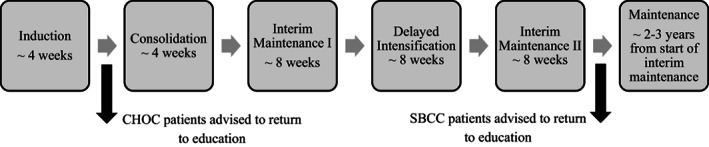
Potential center differences in educational attendance for children treated according to a sample standard risk B‐cell ALL protocol

There appeared to be a difference in the standard advice provided by SBCC and CHOC regarding the safe timing (related to infection risk) of return to social activities and education following an ALL diagnosis. Anecdotal evidence suggested that CHOC families were advised that their child could return immediately after induction therapy while SBCC families were advised to wait until the start of maintenance treatment. Figure 1 illustrates that for children being treated according to a sample standard risk B cell ALL protocol this potentially meant that there was a 28 week period in which a CHOC patient was attending school or early childhood education (ECE) while their counterpart at SBCC was not.

The differences in standard advice between New Zealand's two specialist centers provided the opportunity to address a gap in the international literature regarding the safe timing for children undergoing treatment for ALL to return to education and social activities and to use the results of the study to inform nationally consistent guidelines for New Zealand's two specialist centers. Initially, our study was conceptualized as a simple retrospective review of patient medical records to determine if those treated at CHOC—who were advised to return to education at the end of induction—had a greater number of infections that those treated at SBCC—who were advised to return to education at the start of maintenance. However, at the study design phase it became evident that a medical record review alone would not address firstly, if the many individuals who provided infection control and return to social activities advice to patient families were consistent in the advice that they provided and secondly, if families followed the advice that they received. That is, did children return at the time that families were advised that it was medically safe for them to do so? The study was therefore expanded to become a mixed methods study which incorporated an online survey of healthcare and other professionals who advise families about infection risk and returning to school activities; a retrospective review of both the medical and educational attendance records for children diagnosed with ALL in each center; and in‐depth qualitative interviews with a representative sample of the children's families. These three study components were undertaken concurrently.

Here, we focus on two of the study objectives; whether returning to education prior to the start of maintenance therapy was associated with higher rates of infection in children with ALL; and how families perceive and act on the advice related to their child's infection risk and return to social activities including education.

## 2 METHODS

### 2.1 Study cohort

The study's cohort of children aged 2–13 years who were diagnosed with B‐ or T‐cell ALL in New Zealand between January 2014 and December 2016 was identified through the New Zealand Children's Cancer Registry. At the time the data were collected the children were at different stages of treatment, ranging from maintenance therapy to 1 year off treatment. Those who were deceased, no longer resident in New Zealand or who had recently relapsed were excluded from the study resulting in 97 families being approached to participate in the research. Of the 47 families (48%) that agreed for their child's medical and educational records to be reviewed, 41 also expressed a willingness to participate in a qualitative interview from which 24 families were selected (25% of the total cohort). Quota sampling ensured that those families who were selected to be interviewed were representative of the total cohort in terms of their child's age at diagnosis, treatment center, sex, and ethnicity (see Table 1).

**TABLE 1:** Recruitment target numbers and final numbers for parent interviews

	Total cohort eligible for study		Agreed to participate in study		Families interviewed	
	*n*	(%)	*n*	(%)	*n*	(%)
Sex						
Male	56	(58)	24	(51)	13	(54)
Female	41	(42)	23	(49)	11	(46)
Ethnicity						
New Zealand Māori	18	(19)	6	(13)	5	(21)
Pacific	12	(12)	3	(6)	2	(8)
All Other	67	(69)	38	(81)	17	(71)
Treatment centre						
Auckland—SBCC	32	(33)	11	(23)	6	(25)
Shared care—affiliated with SBCC	26	(27)	12	(26)	7	(29)
Christchurch—CHOC	16	(16)	8	(17)	4	(17)
Shared care—affiliated with CHOC	23	(24)	16	(34)	7	(29)
Education level at diagnosis						
Pre‐school (2–4 years)	54	(57)	29	(62)	12	(50)
School age (5–13 years)	42	(43)	18	(38)	12	(50)
Total	97	100	47	100	24	100

Abbreviations: CHOC, Children's Hematology Oncology Centre; SBCC, Starship Blood and Cancer Centre.

For the medical and educational records review participation rates were higher for families treated by CHOC (24 out of 39 families contacted; 62%) compared to those treated in New Zealand's larger specialist center at SBCC (23 out of 58 families contacted; 39%). In addition, participation rates were lower for Maori and Pacific families, although this was able to be addressed through quota sampling for the qualitative component of the study. Aside from this, the study participants were largely representative of the total cohort and similar across the two centers according to a range of characteristics including sex, involvement with a shared‐care center, enrolment in a clinical trial, risk group, level of deprivation, and family size.

The median age of the study participants at the time of their diagnosis was 3 years and 9 months, reflecting the age at which the incidence of ALL peaks.^1^ Eighteen children (38%) were of school age (5 years or older) at the time of their diagnosis, with the remainder attending some form of formal ECE. In New Zealand, the cost of ECE for those aged 3 years or older can be fully subsidized for up to 20 hours a week and there is no charge for primary school education.^15^ According to Statistics NZ data, in 2017 over two‐thirds of 2‐year‐olds were enrolled in ECE, increasing to 89% of 4‐year‐olds.^16^

### 2.2 Medical and educational records review

A retrospective chart review of medical records in combination with a review of each child's educational attendance records was undertaken in order to determine whether the timing of return to education was associated with an increase in the number of infections during the treatment period between the end of induction and the start of maintenance therapy. While some of these questions could potentially have been answered by the parent, the literature indicates that parental memory is not a reliable record for educational attendance or medical illness.^17^

A password‐encrypted spreadsheet containing the initial demographic and diagnostic data was provided by the New Zealand Children's Cancer Registry. This was then supplemented with relevant data collected by a member of the research team—a medical oncologist—from the medical records on site at the two specialist centers. Infection was defined as any infection recorded in the participant's medical record that resulted in a delay or change to the child's treatment plan. The medical records provided dates of notified infections and the phase of treatment which the child was currently in at the time of the notified infection. Base dates included the date of diagnosis, the date of the end of induction therapy, the date of the start of maintenance, and the date of the end of treatment (where treatment was already completed).

Educational attendance and absence record keeping is a Ministry of Education requirement for all schools and ECE centers. The researchers contacted individual schools and ECE centers to request a copy of the participant's educational attendance records. This included a letter with a brief explanation of the study and a copy of the signed parental consent form for their child's records to be released. The attendance records for five participants could not be obtained from their respective school/ECE. The records obtained for the other 42 participants were from date of diagnosis until the last school day for 2017. The educational attendance records provided the date a participant first returned to school/ECE following diagnosis and a tally of any half days attended between the date of end of induction therapy and the start of maintenance.

Following the retrieval of data from the educational and medical records, all variables were coded and merged onto a single spreadsheet. As the period between the end of induction therapy and the start of maintenance was when the return‐to‐school advice potentially differed between the two centers, this was the specific time‐period that was investigated. Data were analyzed to determine whether the infection rates at the specialist centers differed and whether return to education prior to the start of maintenance was associated with a greater risk of infections. Planned comparisons were conducted to compare the rate of “early” return to school by specialist center and the average number of half days that children attended between the induction therapy and start of maintenance. All statistical analysis was conducted using Stata version 14.

### 2.3 Family interviews

Twenty‐four semi‐structured interviews were conducted with families by telephone by one member of the research team using open‐ended questions based on the interview schedule (see Supplementary Material). The interview schedule was reviewed by a New Zealand consumer representative for child cancer families, a family support coordinator from a child cancer NGO and a pediatric oncologist prior to the study commencement. Interviews were digitally audio recorded and transcribed and ranged from 31 to 132 minutes long, with a median length of 56 minutes.

A deductive thematic analysis approach was used to code relevant interview data chunks. Following the initial coding of 81 descriptive categories, thematic networks were used to determine how they interrelated and to create 14 initial themes.^18^ A second member of the research team reviewed a sample of five of the 24 interviews (21%) to assess inter‐rater reliability.^19^, ^20^, ^21^ This showed 83% alignment across 243 identified themes from the sample interviews, which is well above the 70% considered “acceptable.”^19^ Together the two researchers undertook a process of inter‐coder agreement by re‐working the categories and themes in collaboration to reach consensus.^19^ The final result was 79 categories and 11 themes, which were further distilled down to three overarching themes: consistency, balance, and mental health and wellbeing.

## 3 RESULTS

### 3.1 Medical records review

The overall number of infections recorded between the end of induction and start of maintenance was low, with 64% not developing a single infection that resulted in a deviation from their treatment plan during the study period. The mean per patient infection rate was 0.4 for CHOC patients and 0.7 for SBCC (*P* = .48, see Table 2). Those children who returned to education prior to the start of maintenance therapy were at no greater risk of infection (mean = 0.5) than those who did not return until the start of maintenance (mean = 0.4, *P* = .74). Treatment according to a high‐risk protocol was the only variable examined which was associated with a higher rate of infections (mean = 1.2, *P* = <.01).

**TABLE 2:** Rate of major infections between the end of induction and the start of maintenance therapy for 47 children diagnosed with acute lymphoblastic leukemia in New Zealand, 2014 to 2016

	Participants	Number of infections			
		Mean (SD)	Median (IQR)	Range	*P*‐value
Specialist cancer center					.48
CHOC	24	0.4 (0.7)	0 (0–1)	0–2	
SBCC	23	0.7 (1.0)	0 (0–1)	0–3	
Age at diagnosis					.90
Pre‐school age (2–4 years)	29	0.6 (0.9)	0 (0–1)	0–3	
School age (5–13 years)	18	0.6 (0.9)	0 (0–1)	0–3	
Sex					.32
Female	23	0.4 (0.7)	0 (0–1)	0–2	
Male	24	0.7 (1.0)	0 (0–1)	0–3	
Usual place of residence					.70
Auckland or Christchurch	19	0.5 (0.7)	0 (0–1)	0–2	
Outside of specialist centers	28	0.7 (1.1)	0 (0–1)	0–3	
Level of deprivation					.10
Least deprived	24	0.6 (1.0)	0 (0–1)	0–2	
Average	9	1.0 (1.0)	0 (0–1)	0–3	
Most deprived	14	0.3 (0.6)	0 (0–1)	0–2	
Ethnicity					.47
Māori	6	0.2 (0.4)	0 (0–1)	0–1	
Pacific Peoples	3	0.3 (0.6)	0 (0–1)	0–1	
All Others	38	0.6 (0.9)	0 (0–1)	0–3	
Risk group at diagnosis					<.01
High	13	1.2 (1.1)	1 (0–2)	0–3	
Average	26	0.2 (0.5)	0 (0–0)	0–2	
Low	8	0.5 (0.8)	0 (0–1)	0–2	
Returned to school prior to maintenance?^a^					.74
No	13	0.4 (0.7)	0 (0–1)	0–2	
Yes	29	0.5 (0.9)	0 (0–1)	0–3	

Abbreviations: CHOC, Children's Hematology Oncology Centre; IQR, inter‐quartile range; SBCC, Starship Blood and Cancer Centre; SD, standard deviation.

^a^ School attendance records for five patients were not able to be retrieved and therefore they were excluded from this section.

### 3.2 Educational attendance record review

This study was based on the belief that children undergoing treatment for ALL at CHOC were being advised to return to education after induction therapy while children treated at SBCC were being advised to wait until the start of maintenance. We would therefore have expected that most children treated at CHOC would have returned to school/ECE prior to maintenance therapy and conversely very few of the SBCC patients would have. However, an analysis of the educational attendance records showed there was no statistically significant difference between the two groups in the time period under investigation. Nearly one in four children (23%) who were treated at SBCC *did* return to school/ECE at some point prior to maintenance and although the return figure was higher for children treated on CHOC (40%, *P* = .23), this still means that the majority (60%) of CHOC patients *did not* return to school/ECE at all during this period.

The mean number of half days attended for SBCC patients was 2.1 days compared to CHOC patients was 17.6 days (*P* = .12, see Table 3). This non‐statistically significant difference was largely due to two clear outliers. One CHOC child attended school for 88 half days during this period, while another attended ECE for 152 half days.

**TABLE 3:** Rate of return to education and number of half days attended prior to the start of maintenance therapy for children diagnosed with acute lymphoblastic leukemia in New Zealand, 2014 to 2016

Treatment center	Returned to education prior to start of maintenance			Number of half days attended			
	Yes *n* (%)	No *n* (%)	*P*‐value	Mean (SD)	Median (IQR)	Range	*P*‐value
Children's Hematology Oncology Centre	8 (40%)	12 (60%)	0.23	17.6 (38.0)	0 (0–18)	0–152	0.12
Starship Blood and Cancer Centre	5 (23%)	17 (77%)		2.1 (5.4)	0 (0–0)	0–24	
Total	13 (31%)	29 (69%)		9.5 (27.3)	0 (0–3)	0–152	

Abbreviations: IQR, inter‐quartile range; SD, standard deviation.

### 3.3 Parent interviews

Twenty‐four parent interviews were conducted using a semi‐structured interview schedule. The parents, who were interviewed, were a representative sample of the entire ALL cohort in terms of their child's gender, ethnicity, age at diagnosis, and treatment location. As the cohort included children diagnosed across a 2‐year period, the parents interviewed were all at different stages of treatment, ranging from maintenance therapy to 1‐year off treatment.

The 24 interviews and analysis provided complex and rich data. Parents talked about the advice they got about *when* and *how* to return, *who* they listened to, *what* went well, and what did not. Here, we focus on the three overarching themes, which were identified: consistency, balance, and mental health and wellbeing.

#### 3.3.1 Consistency

Parents sought and received advice on infection risks and the timing of returning to social activities and education from a wide range of medical and non‐medical sources.

When [he] was first diagnosed his lead oncologist basically said these are the things you can and can't do to keep [him] safe… and if I wasn't sure I'd ask the nurses. And I'd quite often I'd ask our family support coordinator from [Child Cancer Foundation] as well. (Interview 8)

Parents expressed a clear desire for consistency in the advice they received. Yet, many parents indicated that they did not receive consistent advice. This inconsistency was generally seen as a negative, especially when it was evident within groups that parents assumed would be cohesive, such as the healthcare team.

All the nurses and doctors are lovely really. It's just that consistent information. And I think if that was there it would make it easier really. And surely it would make it easier for the shared care nurses if they know what the rules are. (Interview 21)

The CHOC message was: if he feels up to something let him do it and send him to school if he wants to go. But the [NGO] message was: hang back, look after yourselves, do only what you think you can take on. And I found that a little bit confusing because we didn't know whether to send him to school or not. (Interview 14)

Without consistency parents sought other opinions, felt a loss of control, and started to doubt themselves and their decisions. Inconsistency could increase a parent's concern that they might not be being given the best advice, that they have made a poor decision or put their child in danger.

And because they got slightly different advice we did think oh, maybe we're taking her back a bit too much. (Interview 11)

Parents expressed a desire for practical resources such as templates of what to tell their school or ECE provider about how to manage their child's infection risk. These resources were seen as a neutral way to communicate important information to friends, teachers, and other contacts in the wider community.

Maybe [the hospital] could just have a little information thing that you could post [online] or print out or something and then you don't have to feel like you're moaning at people or accusing. (Interview 3)

While acknowledging the need for individual variance, parents indicated that consistent advice would be more helpful as parents could then make decisions based on their own circumstances, but secure in the knowledge of what was the advised course of action.

I think written down on a piece of paper—the main key things—would be really useful because then you could go look at your list and go well, I'm allowed to do that but I don't feel ready to do that or I'm allowed to do that but she's really unwell. (Interview 21)

Ultimately, consistent advice and information would help parents trust in themselves and the decisions they make.

You just want to be confident in the decisions you make. As confident as you can be. You gotta feel like you're doing the right thing for yourself, your child and your family. (Interview 23)

#### 3.3.2 Balance

Once leaving hospital, parents take on the primary responsibility of keeping their child safe from infections while also trying to rebuild family routines. Many parents spoke of how they would carefully weigh up of the risk of developing an infection with the desire to return to a sense of normalcy.

You can make calculated risks. … But there were concessions to keeping him sane and making him feel kind of normal and not cracking down on everything. (Interview 16)

We don't want her to become a child that is constantly worrying… Will I get sick? Can I do this? Can I do that? So we wanted to try and get her into a sense of normality as quickly as was possible. (Interview 13)

When parents weighed up the risks and benefits of their child returning to education and social activities, this was always done in consideration of the needs of the entire family.

And for us it was just about a really slow integration, not just for her but for me, to get back into something sort of normal. (Interview 1)

You've just got to work out what is going to work for him and our family. (Interview 23)

While infection risk factored heavily in parental decision making regarding the when and how of returning to education, the child's age, personality, tiredness, and anxiety were also common considerations.

I think over the nine months of intensive treatment he just became less energetic and really didn't want to go to school and we didn't push it. (Interview 11)

We thought that was the best option for her… She was very ready to go back to school and we felt that she was healthy enough. And I have never regretted that decision at all. (Interview 9)

A clear message many of the families recalled getting from their healthcare team was to “return to normal.” Going back to school/ECE was seen as an important part of the returning to normal process and was anticipated by parents with both excitement and trepidation.

To get her back into civilisation. Like be normal, because that's what normal kids do is go to kindy, or daycare, or whatever. (Interview 1)

In New Zealand, children whose health prevents them from regularly attending primary school are eligible for admission to a Regional Health School. It was notable that while parents often spoke positively about the health schools, they did not consider their child's attendance as signifying a return to social or educational activities.

And I think it's that whole normalisation. Trying to make a really abnormal situation as normal as possible. And school was a normality for us… Northern Health School were fantastic but it's still not the same as being at school. (Interview 17)

#### 3.3.3 Mental health and wellbeing

Although it may be expected that having a child undergoing cancer treatment would impact a parent's mental health and wellbeing, the interviews illuminated many areas where parents could have been better supported. Parents spoke often of the emotional work that they did over the course of their child's treatment. This emotional work usually involved the constant vigilance of trying to keep their children safe from infections and providing on‐going communication.

The school would ring us… they'd say there's a case of chickenpox in Room 6. And my next call would be to the outreach nurse, who would then call the paediatrician and they'd help us weigh up the risk. (Interview 17)

Educating family, friends, and others about their child's infection risk was often an additional stressor for parents. Parents expressed a desire for practical resources so that they were not burdened with educating others at a time that they were already physically and emotionally exhausted.

When someone is going back to school or kindy, for there to be like a template or something that's really easy to access to give to the school or kindy. (Interview 10)

If there was someone that could come to the kindy and tell them. Because it gets tiring us relaying information, and I'm sure I miss bits each time. (Interview 8)

All families transitioned between their homes and treating hospital(s) multiple times throughout the course of their child's treatment. Similarly, a clear message from the interviews was that returning to education was not one single moment or date, but took place multiple times, and could be interrupted at any point for any number of reasons including child or family illness, fatigue, and scheduled hospital appointments. These frequent changes—or even the potential for changes—had a clear impact on the parent's mental well‐being, and often their child's as well.

[We'd] just make a bit of progress and get back into the school routine and then he's sick again and it's sort of back to square one again. (Interview 15)

Parents stated that the prolonged period of vigilance and responsibility required when caring for an immunocompromised child placed a burden on the mental health and wellbeing of the whole family, which for some lasted long after their child's cancer treatment had ended.

## 4 DISCUSSION

The medical records review was conducted to address a fundamental gap in the literature on post‐diagnosis experience for families with ALL, namely that there is no empirical evidence to inform the timing of return to education for ALL families.^14^ It was based on anecdotal evidence that different advice was being provided at New Zealand's two specialist centers which afforded us the unique opportunity to undertake a “natural experiment” to determine if there was an increased risk of infection for CHOC patients—who returned to education after induction—compared to SBCC patients who did not return until the start of maintenance. If there was no difference in infection rates between the two groups, then we could confidently advise all New Zealand families with a child undergoing treatment for ALL that it was safe for them to return to education from the end of induction, with the presumptive educational, social, and family benefits which were promoted by this earlier return.^11^, ^12^

Unfortunately, this study not able to conclusively answer the research question on the safe timing of return to education. This is because 60% of CHOC patients did not return to education until the start of maintenance, irrespective of the advice that families may have received from their child's oncologist and others who acted in an advisory role. Our study did, however, find that there was no increased risk of infections for those children who did return to education between the end of induction and the start of maintenance therapy. In addition, the number of infections recorded which resulted in changes to the child's treatment plan were low. In fact, two‐thirds of the children in this cohort did not develop a serious infection according to our study criteria. We hope that this finding can be of some reassurance to those families who are concerned about their child developing an infection once they begin to interact more regularly with others outside of the hospital environment.

The reasons why we were unable to answer our first research question and why the group of children receiving treatment for ALL through CHOC did not return to education any earlier than those from SBCC became apparent through the qualitative component of the study. The 24 parental interviews highlighted two major erroneous assumptions that we had made when we had first conceptualized this piece of research. Firstly, we believed that although the advice around infection control and returning to social activities given to families at each the two specialist centers differed, each family was receiving largely consistent advice from the medical and allied health professionals involved in their child's care. Yet through speaking directly to the families, it became evident that the advice that they were being given was not consistent even from staff at their child's specialist center, let alone between the multitude of others who gave advice to the family over the course of their child's treatment. The inconsistent advice that many families received and the significant negative impact that this had on parental mental wellbeing was a key theme to emerge from the interviews.

Secondly, we wrongly assumed that infection risk was the key consideration when parents made decisions around returning to social activities and education and that parents would be keen for the child to return to social activities as soon as it was declared medically safe for them to do so. However, our parental interviews revealed that infection risk was but one of many factors that these parents considered when navigating their child's “return to normal.” Parents did not make decisions about infection risk and returning to education in isolation, or just with a health focus, but balanced these decisions within the wider context of their lives and their family. Such decisions required them to weigh up financial, emotional, and practical considerations. Their child's personality, response to treatment, and the needs of other family members all featured heavily in their decision making.

Through the parental interviews, it was clear that parents want to make their own decisions about the timing of their child's return to education. Parents make these decisions by carefully considering their child's needs within the context of the needs of the wider family.^22^, ^23^, ^24^ However, to make such decisions an empowering process for the parent, they require clear and consistent information delivered in a way that is tailored to meet the unique needs of their family.^25^, ^26^ Without this, parental perceptions of the health team's capabilities can be reduced.^27^ The inconsistency of advice, the need to educate others and the burden of responsibility were articulated as areas of additional pressure on parents, particularly at a time when parents were already physically and emotionally stretched. This decisional burden and the emotional work in caring for a child with a major health issue can negatively impact parental mental health.^28^, ^29^, ^30^, ^31^, ^32^ An important way for parents to feel a sense of agency is to provide them with information and advice which allows them to make informed decisions.^24^ Our research indicated a clear role here for cancer health and support services to support parents' and children's mental health and wellbeing through providing clear and consistent advice to families. Ultimately, consistent advice and information will help parents trust in themselves and the decisions they make while also giving them greater trust in their health team.^31^, ^32^

**FIGURE 2:**
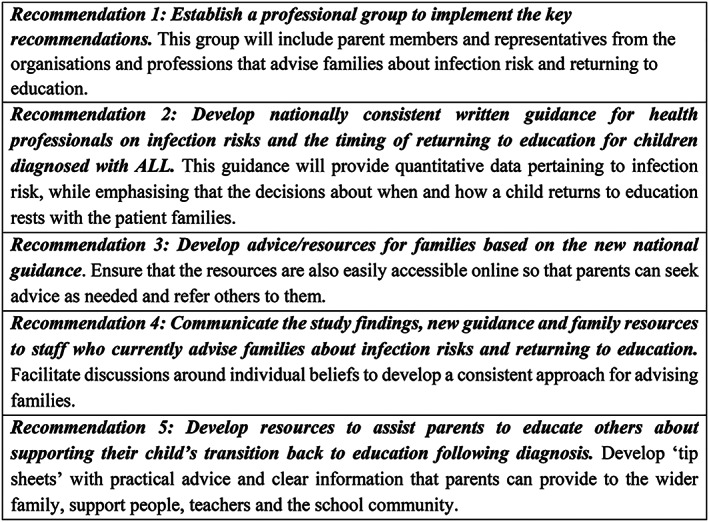
Study recommendations

Following the study conclusion, five major recommendations were made to ensure that the key study findings were addressed (see Figure 2). The National Child Cancer Network established a working group—which includes medical, NGO, educational, and family representatives—to implement the study recommendations. This is the first time that parents have been included in a National Child Cancer Network working group and emphasizes the important role that families can play in ensuring the research findings are successfully translated into practice. The working group will develop a range of practical, user‐friendly resources both for the parents themselves and for them to share with friends, family, school, and their wider social circle. These resources will include direct “parent to parent” advice, which incorporates quotes taken directly from the parent interviews. The new health professional guidelines will provide quantitative data pertaining to infection risk, while emphasizing that the decisions about when and how a child returns to education and social activities rest with the patient families. All health professionals and others who advise families about infection risk and/or returning to education and social activities will be provided with these guidelines to help ensure that the future cancer families receive consistent advice and support during their “return to normal.”

Our original study was conceptualized as a single analysis of infections recorded on patient medical records to identify when a child undergoing treatment for ALL could safely return to education and social activities. Yet had we relied solely on the medical records, we would have concluded that it was safe for a child to return after induction therapy but have failed to identify that many children treated on CHOC were not in fact returning to education at this time. We would also not have gained the insight that infection risk was but one of parents' many considerations when navigating their child's “return to normal.” Ultimately, this would have led to the development of national guidelines that were not fit for purpose or widely adopted. Our study illustrates the importance of listening to parents if we are to create policies that are responsive, relevant, and well received.

Although this paper has discussed the findings from research with a specific population—ALL patients and families from New Zealand—we believe that our learnings around the importance of consistency of advice and supporting parental wellbeing have a much wider application across a variety of populations and contexts within child health. Going beyond the perspectives of healthcare providers to learn from parents' lived experiences will add to current bodies of knowledge and potentially offer new opportunities to identify solutions to improve clinical practice. The parents that we interviewed welcomed the opportunity to participate in research and contribute to initiatives that had the potential to improve the experiences of the families that come after them. This concluding quote reflects a commonly expressed sentiment:

If there is anything that we can do to provide information and help out that can improve things for new patients [and] new parents in any way—even one person—it's good.

## CONFLICT OF INTEREST

The authors declare that there is no conflict of interest.

## AUTHOR CONTRIBUTIONS

All authors had full access to the data in the study and take responsibility for the integrity of the data and the accuracy of the data analysis. *Conceptualization*, S.M., C.G., and K.B.; *Methodology*, C.G., K.B., and S. M.; *Investigation*, C.G., K.B., and S.M.; *Formal Analysis*, C.G., and K.B.; *Writing—Original Draft*, K.B., C.G., S.M., and E.R.; *Writing—Review & Editing*, K.B., E.R., C.G., and S.M.; *Visualization*, K.B., and C.G.; *Project Administration*, C.G., E.R., and K.B.; *Funding Acquisition*, S.M.

## ETHICAL STATEMENT

The study was approved by the Northern B Health and Disability Ethics Committee (17/NTB/241). It conformed to all New Zealand National Ethical Standards for Health and Disability Research and Quality Improvement, published by the National Ethics Advisory Committee. Parental consent and child assent were obtained from all study participants.

## Supporting information


**Appendix** S1: Supporting information

## Data Availability

The data that support the findings of this study are available on request from the corresponding author. The data are not publicly available due to privacy and ethical restrictions.

## References

[1] Steliarova‐Foucher E , Colombet M , Ries LAG , et al. International incidence of childhood cancer, 2001–10: a population‐based registry study. Lancet Oncol. 2017;18(6):719‐731. 10.1016/S1470-2045(17)30186-9. 28410997 PMC5461370

[2] Ballantine K , the NZCCR Working Group . The incidence of childhood cancer in New Zealand 2010–2014: A report from the New Zealand Children's Cancer Registry; 2017. http://childcancernetwork.org.nz/wp-content/uploads/2017/08/NZCCR-Child-Cancer-Incidence-2010-2014-Final-Report-1.pdf. Accessed March 4, 2020.

[3] Hakim H , Dallas R , Zhou Y , et al. Acute respiratory infections in children and adolescents with acute lymphoblastic leukemia. Cancer. 2015;122(5):798‐805. 10.1002/cncr.29833. 26700662 PMC4764417

[4] O'Connor D , Bate J , Wade R , et al. Infection‐related mortality in children with acute lymphoblastic leukemia: an analysis of infectious deaths on UKALL2003. Blood J. 2014;124(7):1056‐1061. 10.1182/blood-2014-03-560847 24904116

[5] Katsimpardi K , Papadakis V , Pangalis A , et al. Infections in a pediatric patient cohort with acute lymphoblastic leukemia during the entire course of treatment. Support Care Cancer. 2006;14(3):277‐284. 16270193 10.1007/s00520-005-0884-6

[6] Bakhshi S , Padmanjali KS , Arya LS . Infections in childhood acute lymphoblastic leukemia: an analysis of 222 febrile neutropenic episodes. Pediatr Hematol Oncol. 2008;25(5):385‐392. 18569840 10.1080/08880010802106564

[7] Helms AS , Schmiegelow K , Brok J , et al. Facilitation of school re‐entry and peer acceptance of children with cancer: a review and meta‐analysis of intervention studies: school re‐entry for children with cancer. Eur J Cancer Care (Engl). 2016;25(1):170‐179. 25204197 10.1111/ecc.12230

[8] Child Cancer Foundation . Back to school for students with cancer. https://www.childcancer.org.nz/assets/Uploads/1731-B2SBrochOCT11.pdf. Accessed April 9, 2021.

[9] St Judes Children's Research Hospital . School support. https://together.stjude.org/en-us/for-families/school.html. Accessed April 9, 2021

[10] Leukemia and Lymphoma Society . Learning and living with cancer; 2020. https://www.lls.org/sites/default/files/file_assets/PS36_Learning_%20Living_2020.pdf. Accessed April 9, 2021

[11] Tsimicalis A , Genest L , Stevens B , Ungar WJ , Barr R . The impact of a childhood cancer diagnosis on the children and siblings' school attendance, performance, and activities: a qualitative descriptive study. J Pediatr Oncol Nurs. 2018;35(2):118‐131. 29192538 10.1177/1043454217741875

[12] Af Sandeberg M , Johansson E , Björk O , Wettergren L . Health‐related quality of life relates to school attendance in children on treatment for cancer. J Pediatr Oncol Nurs. 2008;25(5):265‐274. 18648091 10.1177/1043454208321119

[13] Af Sandeberg M , Wettergren L , Björk O , Arvidson J , Johansson E . Does school attendance during initial cancer treatment in childhood increase the risk of infection?: school attendance and risk of infection. Pediatr Blood Cancer. 2013;60(8):1307‐1312. 23596132 10.1002/pbc.24510

[14] Bira K. School attendance and childhood cancer. Doctoral thesis. Creighton University; 2018.

[15] Ministry of Education . Practical information about education for parents and carers. https://parents.education.govt.nt/early-learning/early-childhood-education/20-hours-ece-2/. Accessed April 9, 2021.

[16] Statistics New Zealand . More toddlers in formal early childhood care; 2017. https://www.stats.govt.nz/news/more-toddlers-in-formal-early-childhood-care

[17] Simpson J , Smith A , Ansell P , Roman E . On behalf of the United Kingdom Childhood Cancer Study Investigators. Childhood leukaemia and infectious exposure: a report from the United Kingdom childhood cancer study (UKCCS). Eur J Cancer. 2007;43(6):2396‐2403. 17826085 10.1016/j.ejca.2007.07.027

[18] Attride‐Stirling J . Thematic networks: an analytic tool for qualitative research. Qual Res. 2001;1:385‐405. 10.1177/146879410100100307.

[19] Armstrong D , Gosling A , Weinman J , Martineau T . The place of inter‐rater reliability in qualitative research: an empirical study. Sociology. 1997;31(3):597‐606.

[20] Campbell JL , Quincy C , Osserman J , Pedersen OK . Coding in‐depth semistructured interviews: problems of unitization and intercoder reliability and agreement. Soc Method Res. 2013;42(3):294‐320.

[21] Belotto MJ . Data analysis methods for qualitative research: managing the challenges of coding, Interrater reliability, and thematic analysis. Qual Rep. 2018;23(11):2622‐2633.

[22] Lipstein EA , Brinkman WB , Britto MT . What is known about parents' treatment decisions? A narrative review of pediatric decision making. Med Decis Making. 2012;32(2):246‐258. 21969136 10.1177/0272989X11421528 PMC3756486

[23] Robertson EG , Wakefield CE , Shaw J , et al. Decision‐making in childhood cancer: parents' and adolescents' views and perceptions. Support Care Cancer. 2019;27(11):4331‐4340. 30880372 10.1007/s00520-019-04728-x

[24] Darcy L , Knutsson S , Huus K , Enskar K . The everyday life of the young child shortly after receiving a cancer diagnosis, from both children's and parent's perspectives. Cancer Nurs. 2014;37(6):445‐456. 24406380 10.1097/NCC.0000000000000114

[25] Sisk BA , Mack JW , Ashworth R , DuBois J . Communication in pediatric oncology: state of the field and research agenda. Pediatr Blood Cancer. 2018;65(1):e26727. 10.1002/pbc.26727. PMC6902431 28748597

[26] Selwood K , Hemsworth S , Rigg J . Children with cancer: quality of information for returning to school. Nurs Child Young People. 2013;25(5):14‐18. 10.7748/ncyp2013.06.25.5.14.e176 23988071

[27] Anderson KJ , Bradford NK , Clark JE . Through their eyes: parental perceptions on hospital admissions for febrile neutropenia in children with cancer. J Pediatr Oncol Nurs. 2018;35(5):342‐352. 29871527 10.1177/1043454218777719

[28] Sisk BA , Kang TI , Goldstein R , DuBois JM , Mack JW . Decisional burden among parents of children with cancer. Cancer. 2019;125(8):1365‐1372. 30602060 10.1002/cncr.31939

[29] Watt L . “Her life rests on your shoulders”: doing worry as emotion work in the care of children with diabetes. Glob Qual Nurs Res. 2017;4:2333393617743638. 29242810 10.1177/2333393617743638 PMC5724634

[30] Sim C‐W , Heuse S , Weigel D , Kendel F . If only I could turn back time‐regret in bereaved parents. Pediatr Blood Cancer. 2020;67(6):e28265. 32196890 10.1002/pbc.28265

[31] Rafferty KA , Hutton K , Heller S . “I will communicate with you, but let me be in control”: understanding how parents manage private information about their chronically ill children. Health Commun. 2019;34(1):100‐109. 29072494 10.1080/10410236.2017.1384432

[32] Ruble K , Paré‐Blagoev J , Cooper S , Martin A , Jacobson LA . Parent perspectives on oncology team communication regarding neurocognitive impacts of cancer therapy and school reentry. Pediatr Blood Cancer. 2019;66(1):e27427. 30160071 10.1002/pbc.27427

