# Supplementary material for: Listening to the experts: Parents' perspectives around infection risk and returning to education and social activities following their child's diagnosis of acute lymphoblastic leukemia
Source: Cancer Rep (Hoboken). 2021 May 14;5(6):e1424. doi: 10.1002/cnr2.1424 (PMC9199503; doi:10.1002/cnr2.1424)
Supplement: Supplementary file 1 — Appendix S1: Supporting information [file CNR2-5-e1424-s001.docx]

**Supplemental Material S1**

**Interview Schedule**

**Family’s experience pre/early diagnosis**

1. Could you tell me a little about your family’s lives just before your child was diagnosed with ALL?

2. What sort of social activities had they done prior to diagnosis?

3. Can you tell me a little about when your child was diagnosed?

4. Who from your family/whānau was/is involved in the day-to-day care of your child when they were in hospital? And once they came home?

**Parental perceptions and practices**

5. How do you think of the different periods in your family’s experience with ALL?

6. How did you feel about infection risks when your child left hospital and returned home?

7. Thinking about infection risks, did you/your family change your behaviour and household practices post-diagnosis?

8. Were there particular transitions/times that were the most significant to you around infection risks?

**Sources of information**

9. Who gave you information about infection risks?

10. Who gave you information about returning to social activities following diagnosis?

**The information given**

11. Did you get the information when and how you wanted it?

12. What infection prevention advice were you given?

13. Did they talk to you about why it was important to return to social activities?

14. Did they talk to you about potential difficulties about returning to social activities?

**How the information was used**

15. How did you prefer to get your information?

16. Who did you prefer to get the information from?

17. Did you follow up from advice given my medical staff with other sources?

18. Whose advice did you rely on most?

19. Did you follow the advice given?

20. Were there some circumstances that led you to not follow the advice?

**Family’s experiences going back into school/ECE/social activities**

21. What was your experience of your child returning to social activities following diagnosis?

22. What were the main things you considered when deciding when to return to social activities following diagnosis?

23. Can you tell me about what it was like, for your child and your family when your child returned to school/ECE?

24. Did you follow the advice you had been given about timing of returning to school/ECE?

25. Did you think about returning to/starting school/early childhood differently from returning to other social activities?

26. Who organised/helped manage the return to/start of school?

27. Did you do a graduated return?

28. How was the school/kindergarten/creche?

29. Who did you deal with mostly?

30. Under what instances did you/would the school get in contact with you?

31. What systems were in place for you to be told if there were any issues from school (such as sickness in the school community)?

32. Did you have to bring your child out of school because of infection risks?

33. Did you bring siblings out of school for infection risks?

34. Do you think the school/ECE was adequately prepared for your child’s return/start?

**Future/Changes**

35. Is there anything you would have liked done differently in terms of the advice you received?

36. Is there anything you would have liked done differently in terms of returning to social activities/school/ ECE?

37. Is there anything you feel the advice you had received didn’t prepare you for, when returning to social activities?

38. Is there anything else you would like me to know, or you would like to talk about?
